# A Passive but Grave Menace

**Published:** 1919-09-27

**Authors:** 


### FOOD CONDITIONS IN GERMANY.

### I. A Passive but Grave Menace.

A most important Report on food conditions in Germany has just been issued in the form of a White Paper. It is by Ernest H. Starling, C.M.G., M.D., F.R.S., with memoranda on Agricultural Conditions in Germany by A. F. McDougall, Chief Live Stock Commissioner for Scotland, and on Agricultural Statistics by C. W. Guilleband, M.A., Fellow of St. John's College, Cambridge.

Professor Starling and his coadjutors paid two visits to Germany, the first to Cologne and the Rhine district, and the second to Berlin and Kattowitz in Upper Silesia. Before entering into a detailed account of the findings of the Commission, the feeling which one has on reading the Report can best be expressed in Prof. Starling's words: "The impression we have derived is that the nation of Germany is broken, both in body and spirit," that "it will take one and perhaps even two generations before she can recover her previous efficiency," "after this whether she is a danger or not to Europe depends on her Government," and that "the docile and industrious people are at any rate sickened of war, and represent no longer any active menace to the peace of Europe." Certainly no active menace, but, as the Report shows, if she is not assisted by food and capital to recover herself she may prove a very grave passive menace should she be irretrievably ruined by famine.

### Her Home Production.

The food problem in Germany will be best understood if one keeps clearly in mind that the population of Germany, which before the war was 67 millions, was for rationing purposes divided into one-third food producers, and two-thirds food consumers, and that 85 per cent. of her food was produced within her own borders, and 15 per cent. was imported. This, however, does not truly represent her independence of imported foods, since this high figure for home production was only attained by an enormous importation of artificial manures—phosphates and saltpetre—and concentrated feeding stuffs for cattle, as well as the application of every known scientific method of raising the productivity of German soil, which is much poorer than that of England, and consists mostly of light sand "the fertility of which can only be maintained by heavy applications of manure, both farmyard and chemical."

### The Effects of Blockade.

The result of this was that when the blockade became really effective in the spring of 1916, it meant not only the cutting off of the 15 per cent. imported food, but the cutting down of home production, by depriving the farmer of a large amount of chemical manure, as well as concentrated feeding-stuffs for his cattle. This state of affairs was of course aggravated by the diversion of the ammonia and nitrates produced in the country to the production of explosives, the reduction in offals for feeding cattle by milling the grain for human food to 94.97 per cent, instead of the pre-war 70 per cent. As this compulsory neglect of the soil has continued for about three years, it will take some time before its productivity can be raised to pre-war standard.

### Rationing as it Was.

The second great difficulty the Germans had to contend with was rationing. Practically all foods were rationed, but though the producers—*i.e.*, one-third of the population—were rationed more liberally than the consumers, they kept to the pre-war habits and consumed their 4,000 calories a day, leaving only 2,065 per head for the consumers, and this even they did not get, since 25 per cent. of the surplus was disposed of by producers by illicit trading, leaving only some 1,500 calories for those of the population—*viz.*, all but about 2 or 3 per cent.—who were too poor to take advantage of this. Since the physiological needs of a man doing sedentary work are 2,400 calories per diem, it is easy to see that some two-thirds of the population are enduring slow starvation, which if not arrested can only end in acute widespread famine and disease.

### Uneven Distribution.

The third great difficulty with regard to food was and is the controlling of supplies so as to ensure even distribution. As we have seen, the producers refused to ration themselves, but not only this, as they consisted of large and small farms all over the country, it was impossible not only to keep an account of their production, but to prevent them concealing it and disposing of a large percentage by illicit trading. On the other hand, as so large a proportion of our own food is imported, the Government could obtain control at the port of entry and see that the distribution was equitable.

### Illicit Trading.

This illicit trading was one of the most serious difficulties, since the rich and the war profiteers were willing to pay any price to satisfy their appetites, and the case is mentioned of one family, which spent pre-war 25,000 marks per annum on food and servants, paying 250,000 marks per annum for the same objects. To make matters worse, this illicit trading was carried on not only by individuals but by large firms, such as Scherings in Berlin, with the connivance of the Government, to supplement the rations of their workpeople.

In the next article we propose dealing with the medical aspects of the insufficiency of the diet in Germany.

